# Genes Common in Primary Immunodeficiencies and Cancer Display Overrepresentation of Codon CTG and Dominant Role of Selection Pressure in Shaping Codon Usage

**DOI:** 10.3390/biomedicines9081001

**Published:** 2021-08-12

**Authors:** Rekha Khandia, Taha Alqahtani, Ali M. Alqahtani

**Affiliations:** 1 Department of Biochemistry and Genetics, Barkatullah University, Bhopal 462026, India; 2 Department of Pharmacology, College of Pharmacy, King Khalid University, Abha 62529, Saudi Arabia; ttaha@kku.edu.sa (T.A.); amsfr@kku.edu.sa (A.M.A.)

**Keywords:** CTG overrepresentation, primary immunodeficiencies and cancer, gene expression level, natural selection

## Abstract

Primary immunodeficiencies (PIDs) are disorders of the immune system that involve faulty cellular, humoral, or both cellular and humoral functions. PIDs are present at the crossroad between infections, immune dysregulation, and cancers. A panel encompassing 42 genes involved in both PIDs and cancer has been investigated for the genes’ compositional properties, codon usage patterns, various forces affecting codon choice, protein properties, and gene expression profiles. In the present study, the codon choice of genes was found to be dependent upon the richness of the nucleotide; the viz AT nucleotide rich genome preferred AT ending codons. The dinucleotide TpA adversely affected protein expression, while CpG did not. The CTG codon was the most overrepresented codon in 80.95% of genes. Analysis of various protein properties, including GRAVY, AROMA, isoelectric point, aliphatic index, hydrophobicity, instability index, and numbers of acidic, basic, and neutral amino acid residues revealed that the hydrophobicity index, instability index, and numbers of acidic and basic amino acid residues are the factors affecting gene expression. Based on neutrality analysis, parity analysis, ENc-GC3 analysis, and regression analysis of nucleotides present at the first and third positions of the codon, it was determined that selection pressure, mutation pressure, and compositional constraints all participated in shaping codon usage. The study will help determine the various evolutionary forces acting on genes common to both PIDs and cancer. Codon usage analysis might be helpful in the future to augment both diseases simultaneously. The research also indicates a peculiar pattern adapted by a set of genes involved in any disease.

## 1. Introduction

A fully functional immune system is a prerequisite to fight against infections and surveil cellular transformations like cancer development. Primary immunodeficiency diseases (PIDs) encompass a heterogeneous group of heritable genetic disorders into which the immune system is partially or fully non-functional [1]. PIDs may culminate in increased susceptibility to infections, autoimmunity, inflammatory organ damage, and malignancy [2]. Initial evidence for linkages between cancer and primary immunodeficiencies (PIDs) was first reported in 1958 [3,4]. Technological intervention in next-generation sequencing made genome analysis possible at a large level and made the PID genetics analysis faster. So far, more than 350 PID-causing genes have been reported [5]. In patients with PID, an enhanced risk of malignancy is reported. There is at least a 1.6-fold greater risk of malignancies in PID patients in comparison to the general population [6], and lymphomas [7] and skin tumors are the commonest ones [8]. Malignancy is considered as a diagnostic marker in a few PIDs [European Society of Immunodeficiencies (ESID) (https://esid.org (accessed on 26 June 2021))] and is the second leading cause of mortality in PID patients [2]. The information regarding the genetic basis of PIDs and malignancy predisposition is chiefly based on monogenic disorders. A single or similar phenotype will be produced by different genes [9]. Neven et al. (2013) reported molecular and immunophenotypical resemblance between the lymphomas in patients with IL10 and IL10R loss-of-function mutations (responsible for early-onset of inflammatory bowel disease) and germinal center B-cell diffuse large B-cell lymphoma [10].

A visualization of the intersection of PID-causing genes (https://esid.org (accessed on 26 June 2021)) with true cancer genes (https://cancer.sanger.ac.uk/census (accessed on 26 June 2021)) and cancer predisposition genes [11] by Derpoorter et al., (2018) revealed a panel of 42 genes that encompass genes involved in defects in innate immunity, antibody disorders, phagocytic disorders, combined immunodeficiency, and immune dysregulation, and common to cancer [2]. To understand the genetic architecture of genes involved in PID, in the present study, various features of these genes like compositional parameters, expression level, protein indices, relative synonymous codon usage (RSCU), and codon usage bias (CUB), and the effects of various evolutionary forces like selectional, mutational, and compositional ones were calculated along with the determination of their inter-relationship. The study will help in investigating the genetic architecture of the genes common to both PIDs and cancer. Elucidation of genetic architecture in the future might be helpful to obtain a common therapeutic substance addressing both diseases simultaneously. Also, the study will help envisage the evolutionary forces responsible for shaping codon usage.

## 2. Materials and Methods

### 2.1. Data Collection

Sequences of 42 genes (Table 1) common to both PIDs and cancer in human beings were retrieved from the National Center for Biotechnology Information (NCBI), U.S. National Library of Medicine nucleotide database. Sequences in the open reading frame and divisible by three were included, and those with internal stop codons or bases other than A, T, C, and G were omitted.

### 2.2. Compositional Analysis

The overall %A, %T, %G, and %C were analyzed, and %A3, %T3, %C3, and %G3 (nucleotide occurrence at the third codon position) were also evaluated and used for parity analysis. The average %GC12 (G/C present at the first and second positions) and %GC3 (G/C present at the third position) were calculated to obtain neutrality. Informatics software CodonW 1.4.4, (an open source software by John F. Peden) available at http://codonw.sourceforge.net (accessed on 26 June 2021), was used for these calculations.

### 2.3. Dinucleotide Abundance

The odds ratio (observed to expected frequency of dinucleotides) was obtained for 42 genes using DNASTAR Lasergene Inc. software (https://www.dnastar.com/software/lasergene/ (accessed on 28 June 2021)). An odds ratio below 0.78 is considered underrepresented and above 1.23 is considered overrepresented [12].

### 2.4. Relative Synonymous Codon Usage (RSCU) Analysis

There are 64 codons (nucleotide triplets) in the standard genetic code, three of which (TAA, TAG, TGA) are stop codons. The remaining 61 codons encode for the 20 standard amino acids [13]. Except for methionine and tryptophan, all amino acids are encoded by two or more than two triplets, and such codons are called synonymous codons. These synonymous codons are not used in equal proportions by cells. This bias, often observed in codon usage, is referred to as codon usage bias (CUB) or codon usage preferences.

The ratio observed to the expected frequency of a codon coding for a particular amino acid (AA) is its relative synonymous codon usage (RSCU) value. It indicates the frequency with which a codon is used among all codons coding for the same amino acid. The codons with RSCU values below 0.6 are underrepresented, while those above 1.6 are overrepresented [14]. We performed RSCU analysis using CodonW 1.4.4 software (http://codonw.sourceforge.net (accessed on 28 June 2021)). The RSCU values of each gene sharing role in PIDs and cancers are enlisted, and the codon with the highest RSCU value for a given amino acid is highlighted in supplementary Table S1.

### 2.5. Codon Adaptation Index (CAI59 and CAI18)

The CAI value suggests the adaptability of any gene and also indicates the level of expression. Two kinds of CAI values, namely, CAI59 and CAI18, were calculated using COUSIN software developed by Bourret et al. (2019) [15]. CAI59 is the classical CAI value, where the impact of 59 codons is assessed, while in CAI18, the impact of amino acid composition on CUB is estimated. The value of CAI ranges between 0 and 1 [16]. Values closer to 1 are present in highly expressed genes.

### 2.6. ENc Determination

ENc is a non-directional measure of CUB, with values ranging between 20 and 61. The lowest value indicates the highest CUB, and vice versa. A value of 20 indicates usage of only one codon out of several synonymous codons, while a value of 61 indicates equal usage of all the codons encoding for a particular amino acid. ENc values were calculated for all the 42 genes using informatics software CodonW 1.4.4, available at http://codonw.sourceforge.net (accessed on 28 June 2021).

### 2.7. Quantitation of Selection and Mutational Forces

A regression plot between %GC3 and an average of %GC12 was constructed to quantify the selectional and mutational pressures imposed on the genes studied. Regression coefficients represent the equilibrium point between mutation and selection force [17]. Values approaching 1 indicate the dominance of mutational force.

### 2.8. Principal Component Analysis (PCA Analysis)

PCA analysis is a statistical method to determine the most influential principal component and used to determine the two principal components most affecting the codon usage. RSCU values of 59 codons excluding the stop codons (TAA, TAG, TGA), a start codon (ATG), and tryptophan (UGG) were represented by 59 independent vectors, and a plot was drawn between the first and second axes. Biplot analysis was also conducted to determine the most influential codons along the two axes.

### 2.9. Protein Indices Calculation

Different characteristics of proteins have been reported to influence CUB. Various parameters explaining the properties of amino acids such as GRAVY (grand average of hydropathy, encompassing features of both the hydrophobicity and hydrophilicity), AROMA (aromaticity—the frequency of aromatic amino acids, i.e., Phe, Tyr, and Trp, in a given amino acid sequence), isoelectric point (PI), aliphatic index (AI), hydrophobicity index (HY), instability index (INSTAB), and the numbers of acidic amino acid residues (acidic AA), basic amino acid residues (basic AA), and neutral amino acid residues (neutral AA) were determined. GRAVY protein values range between −2 and +2, with a positive value indicating a more hydrophobic protein nature, and vice versa [18]. AROMA refers to the frequency of phenylalanine, tyrosine, and tryptophan (all aromatic amino acids) in any protein sequence [19]. Both of these indices are indicative of selection pressure [20]. The hydrophobicity index measures a protein’s solubility and has roles in protein-protein interactions [21]. The instability index indicates protein stability under both in vivo and in vitro conditions, and values of more than 40 indicate unstable proteins [22]. The aliphatic index (AI) refers to the volume occupied by aliphatic side chains, and with the instability index, AI is another parameter to define the stability of the proteins [23]. All the parameters were calculated using Protparam Expasy available at https://web.expasy.org/protparam/ (assessed on 6 July 2021) [24] and PEPTIDE 2 tools (https://www.peptide2.com/) assessed on 6 July 2021.

### 2.10. Statistical Analysis

Correlation analysis was conducted for various parameters, including protein indices, nucleotide positions, and dinucleotide frequencies, and correlation coefficients were calculated and plotted using PAST4 software. Regression analysis was done using PAST4 software [25].

## 3. Results

### 3.1. Compositional Analysis

**Figure 1:**
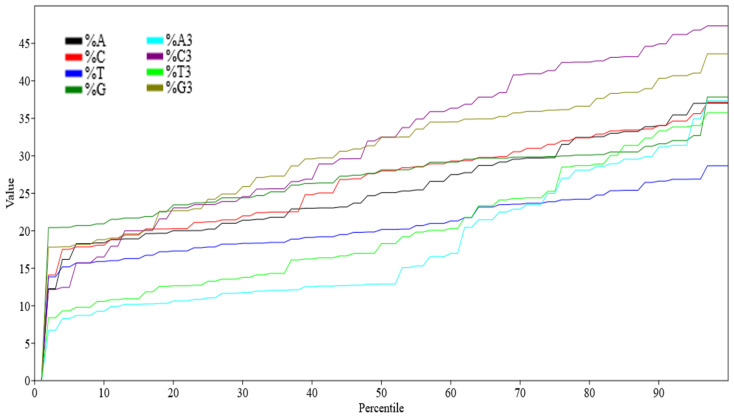
Percentile graph depicting overall nucleotide composition of 42 genes related to SCID.

**Figure 2:**
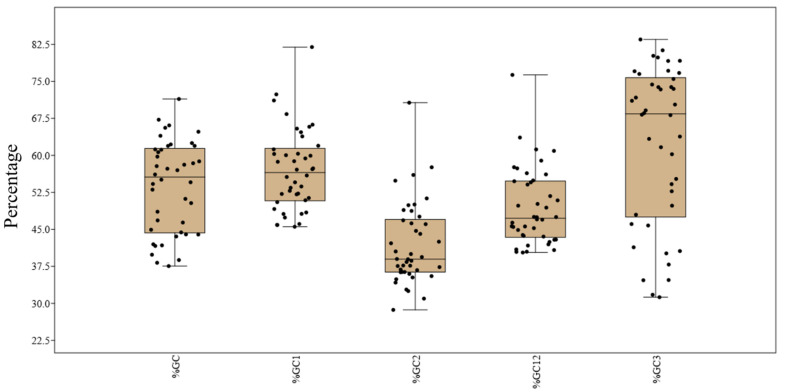
Jitter and boxplot showing percent occurrence of GC components at all positions.

Nucleotide %A values ranged between 12.28% (CDKN2A) and 37.04% (*KRAS*), %C ranged between 14.10% (*KRAS*) and 37.14% (*GATA2*), %T ranged between 13.84% (*TCF3*) and 28.66% (*ATM*), and %G ranged between 20.42% (*BLM*) and 37.84% (*CDKN2A*). Among all the nucleotides, %T representation was the lowest and is depicted by a percentile graph (Figure 1). Percent GC components at all positions (overall %GC, %GC1, %GC2, %GC12, and %GC3) were determined, plotted, and are presented in Figure 2, showing that the maximum variation in composition occurred in %GC3.

### 3.2. Relation of Protein Length on GC12 and GC3 Content

**Figure 3:**
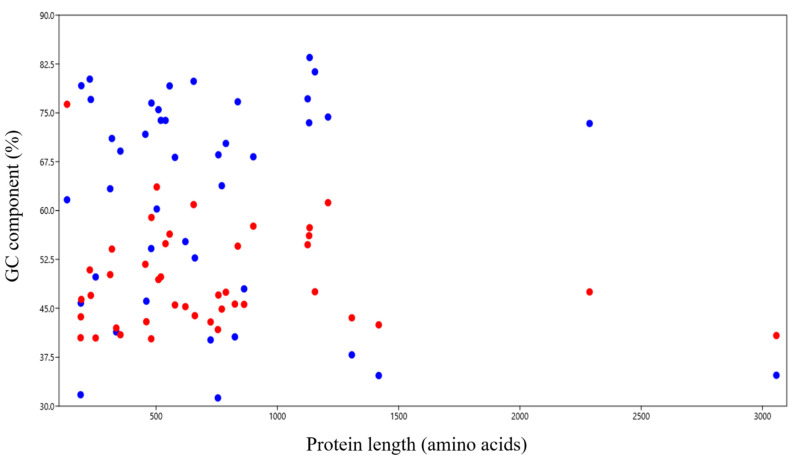
Relationship between protein length and %GC12 and %GC3 content of genes. Blue dots indicate %GC3 and red dots indicate %GC12.

The majority of proteins were smaller than 1500 amino acids. In all the proteins from 150 to 3000 amino acids, GC12 content was lower than GC3 without any exception (Figure 3). The genes below this size (*CDKN2A*) and above this size (*ATM*), had a %GC12 content higher than %GC3 (76.31% and 40.81% for CDKN2A and ATM genes, respectively).

### 3.3. Relationship between GC Component and Gene Expression and CUB

A correlation analysis between overall GC content and GC components at different positions for CAI-59 and ENc was conducted to determine the impact of the GC composition on the gene component and CUB (Table 2). The analysis revealed that gene expression was significantly correlated with components at the GC3 position (r = 0.889, *p* < 0.0001) but did not correlate with either the %GC2 or %GC12 positions.

### 3.4. Relationship between the CUB and Nucleotide Composition with Respect to Codon Position

The effect of the nucleotide position in the codon on CUB was determined by correlation analysis, which revealed all the nucleotides had a significant effect on CUB, except for T1 and G1, which demonstrated that these nucleotides at the first position of the codon had no impact on CUB. Nucleotide T at the second position of the codon (T2) had the least impact (*p* < 0.05), followed by C2 (*p* < 0.01), when compared to all other nucleotides (*p* < 0.001) at different positions of the codon (Table 3).

### 3.5. Dinucleotide Analysis

**Figure 4:**
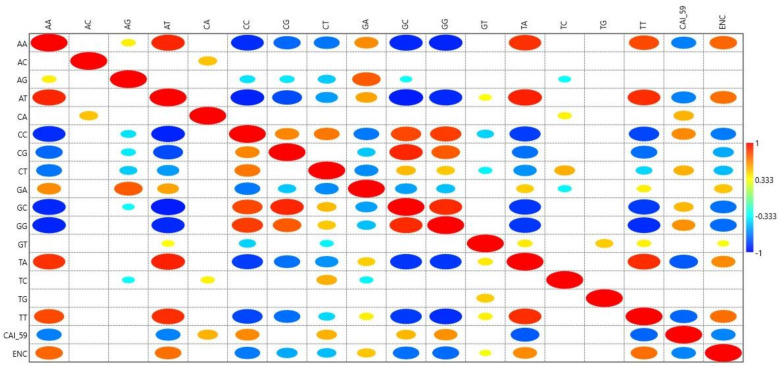
Mirror image plot depicting the correlation between CAI-59 and 16 dinucleotides. A bigger-sized eclipse described a higher Pearson’s correlation coefficient (r) value, and vice versa. The red color shows a positive correlation, while blue indicates a negative correlation. Empty boxes show an insignificant correlation.

The CpG and TpA dinucleotide combination is commonly underrepresented [17,26]. A correlation analysis between the frequency of 16 dinucleotides with CAI-59 revealed that among CpG and TpA, the two nucleotides at those frequencies are strongly driven by selectional force [17]; CpG did not correlate with CAI-59, while TpA negatively correlated (r = −0.770, *p* < 0.0001). CAI-59 correlated negatively with AA, AT, TA, and TT and positively with CA, CC, CT, GC, and GG (r = 0.535, *p* < 0.001; r = 0.643, *p* < 0.001; r = 0.538, *p* < 0.001; r = 0.518, *p* < 0.001 and r = 0.621, *p* < 0.001, respectively). AC and TG dinucleotides positively correlated with their palindromic dinucleotides (CA and GT only). A correlation analysis of the odds ratios with ENc to determine CUB revealed a correlation (*p* < 0.05) in all dinucleotide combinations except for AC, AG, CA, TC, and TG (Figure 4).

### 3.6. RSCU Analysis

**Figure 5:**
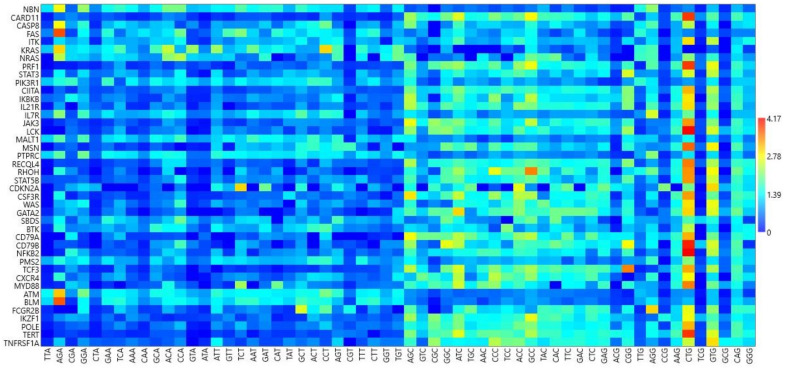
Matrix heat map for RSCU values of codons ending with A, T, G, and G (from right to left).

**Figure 6:**
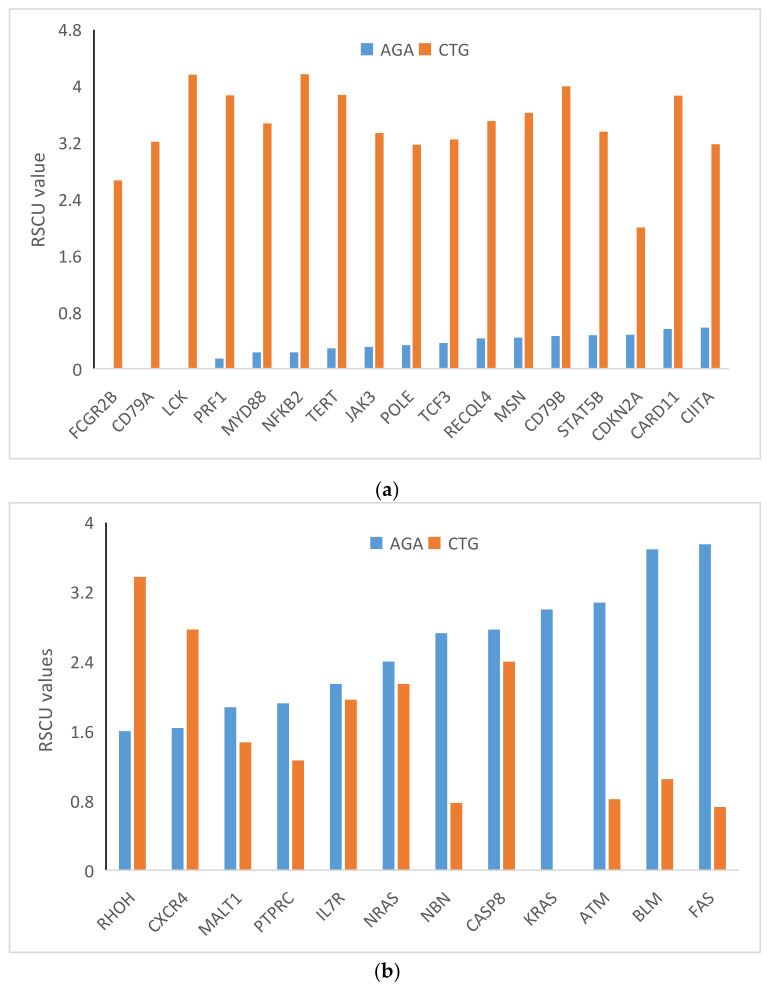
(**a**) Plot of under-represented AGA (RSCU values below 0.6) with corresponding CTG usage. (**b**) Plot of overrepresented AGA (RSCU > 1.6) with corresponding CTG usage.

RSCU analysis of 59 codons (methionine, tryptophan, and stop codons were excluded) indicated low usage of codons ending in A and T while codons ending in C and G were preferred, with high RSCU (Figure 5). Codon CTG was overrepresented in most genes. The CTG codon was overexpressed in all the genes except for a few (overrepresentation [RSCU > 1.6] in 80.95% of genes; underrepresentation [RSCU = 0] in 2.38% of genes; random usage (1.6  >  RSCU  >  0.6) in 16.66% of genes]. Despite general overrepresentation, this codon was not used by the *KRAS* gene (RSCU = 0). The RSCU value was highest for CTG in the *NFKB2* gene (RSCU = 4.17). Plots of underrepresented AGA (RSCU < 0.6) with corresponding CTG usage and overrepresented AGA (RSCU > 1.6) are depicted in Figure 6a,b, respectively. Notably, these results indicate that the AGA codon was underrepresented and the CTG codon overrepresented (Figure 6a,b). A correlation analysis between AGA and CTG revealed a very high negative correlation (r = −825, *p* < 0.0001). However, AGA and CTG were not mutually exclusive, and in a few genes, both AGA and CTG were overrepresented.

### 3.7. Relationship between Gene Expression and Relative Frequency of Codon Usage

**Figure 7:**
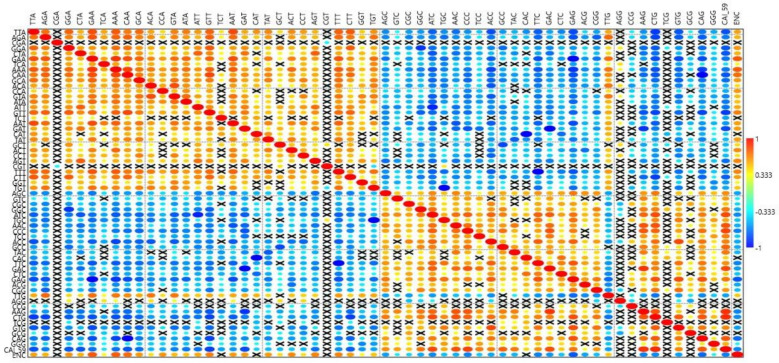
Mirror image plot depicting the correlation between RSCU values with gene expression and CUB. A bigger-sized eclipse described a higher Pearson’s correlation coefficient (r) value, and vice versa. The red colored circles show a positive correlation, whilst blue circles indicate a negative correlation. Crossed boxes show an insignificant correlation.

A correlation analysis was performed between the RSCU values and CAI-59 to elucidate the relationship between the relative frequency of codon usage and gene expression (Figure 7). CGA, CGT, AGG, CCG, TCG, and GCG did not correlate with CAI-59. The CGA codon frequency only correlated with CGT (r = −0.337, *p* < 0.05), while CGT correlated with CGA (r = −0.270, *p* < 0.05), TGT (r = −0.380, *p* < 0.05), TGC (r = −0.381, *p* < 0.05), and GCT (r = −0.405, *p* < 0.05). All other codons correlated with at least ten other codons. All codons ending with CG negatively correlated with codons ending in AT except for TTG and AGG, which positively correlated with codons ending in AT and negatively correlated with codons ending in GC.

### 3.8. Principal Component Analysis (PCA)

**Figure 8:**
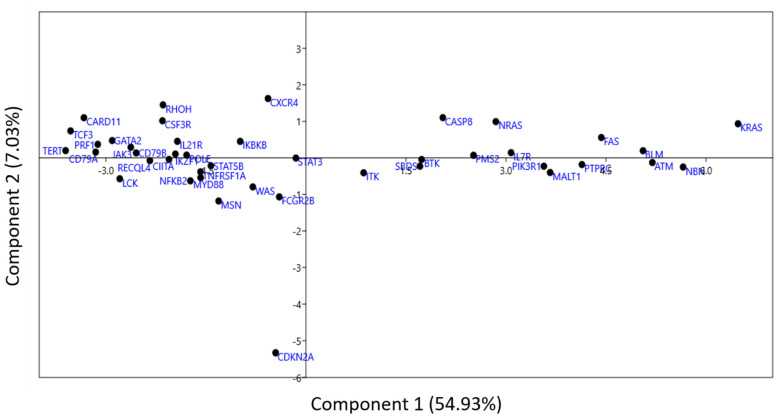
PCA analysis using RSCU values as 59 independent variables. Each dot corresponds to the position of a gene across axis 1 and 2. Each black dot represents a gene. Besides the dot, the name of the gene has been written in blue font.

To further analyze the codon usage patterns in genes common in PIDs and cancers, PCA analysis was performed to determine the distribution of 59 independent variables [27]. The distribution of each vector is depicted in Figure 8. The PCA analysis indicated that the first four axes contributed 54.94%, 7.03%, 5.06%, and 4.08% of all variation, respectively, for a total of 71.11%.

**Figure 9:**
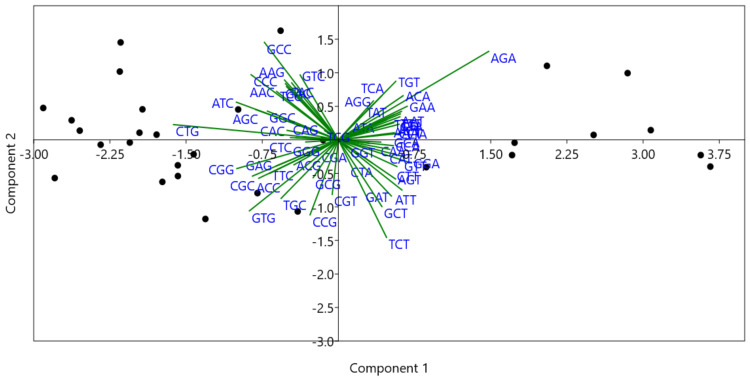
Biplot analysis showing CTG and AGA codons are most influential across axis 1. The length of the arrow is proportional to the influence of the codon on codon usage bias.

Biplot analysis (Figure 9) shows genes and the variable projections to the first two axes simultaneously. The length of the arrow indicates the most influential vectors with the highest loading values [28]. CTG and AGA codons had the highest loading values across PC1, while codons TCT and GCC had the highest loading values across PC2.

### 3.9. Assessment of Compositional Constraints, Selection Pressure, and Mutational Force

**Figure 10:**
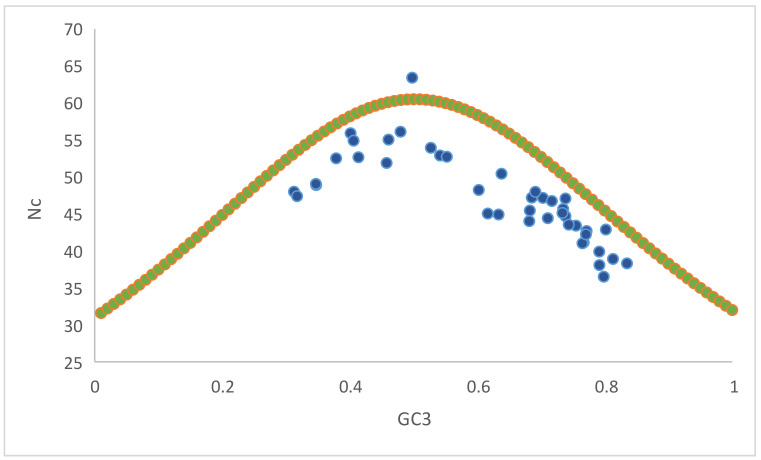
Nc-GC3 curve for determining the compositional constrain selection pressure and mutational force.

The ENc-GC3 plot was constructed to assess the compositional constraints, selection pressure, and mutational forces acting upon genes, determining codon usage. If the data points occur only on the expected ENc line, then the codon usage is driven solely by compositional constraints. The values above the curve indicate mutational forces, while values below the expected curve indicate selectional forces. In the present study, all the values but one were below the expected curve (Figure 10), indicating the presence of selectional forces. One data point above the expected curve explained the presence of mutational forces.

### 3.10. Selection Force Is Dominant over Mutational Force

**Figure 11:**
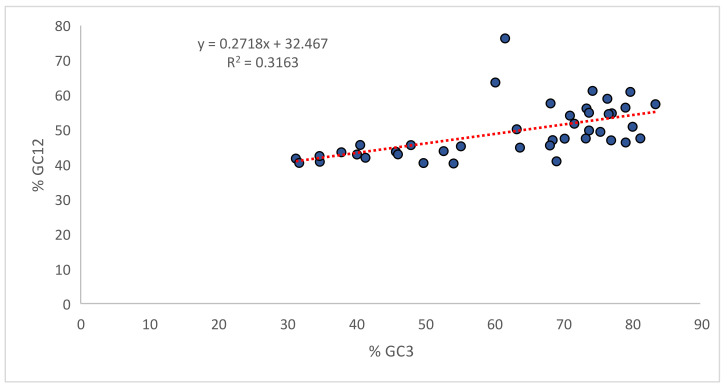
Neutrality plot analysis between %GC12 and %GC3. The slope signifies the impact of mutation and selectional forces.

The %GC3 values ranged between 31.25% to 83.49%, while the average GC12% value ranged between 40.31% and 76.31%. The linear regression model of GC12 on GC3 indicated (GC12%) = 0.2718 (GC3%) + 32.468, with R^2^ = 0.316. This meant that relative neutrality was 27.18%, while the contribution of selectional forces was 72.82%. The plot also indicated that GC3 is responsible for 31.6% of the variation in GC12. (Figure 11) This result shows a high correlation between GC12 and GC3, indicating that the directional mutational force acted on all codon positions [29].

### 3.11. Role of Mutational Force on CUB

Sequence changes can result from mutational pressure, which is one of the imperative factors affecting CUB. The correlation analysis between the overall nucleotide composition of an individual nucleotide and its composition at the third codon position was determined to elucidate how these are related. The correlations are provided in Table 4. Positive correlations were observed between A3-A, A3-T, C3-C, C3-G, C3-GC3, T3-A, T3-T, G3-C, G3-G, G3-GC3, GC3-C, GC3-G, and GC3-GC, hence showing a proportionate dependence. Negative correlations between A3-C, A3-G, A3-GC3, C3-A, C3-T, T3-C, T3-G, T3-GC, G3-A, G3-T, GC3-A, and GC3-T (for all correlations, *p* < 0.0001) represented inversely proportional dependence. Significant correlations among homogeneous nucleotide compositions indicated that the compositional properties attributed to the mutational forces helped shape codon usage [30].

**Figure 12:**
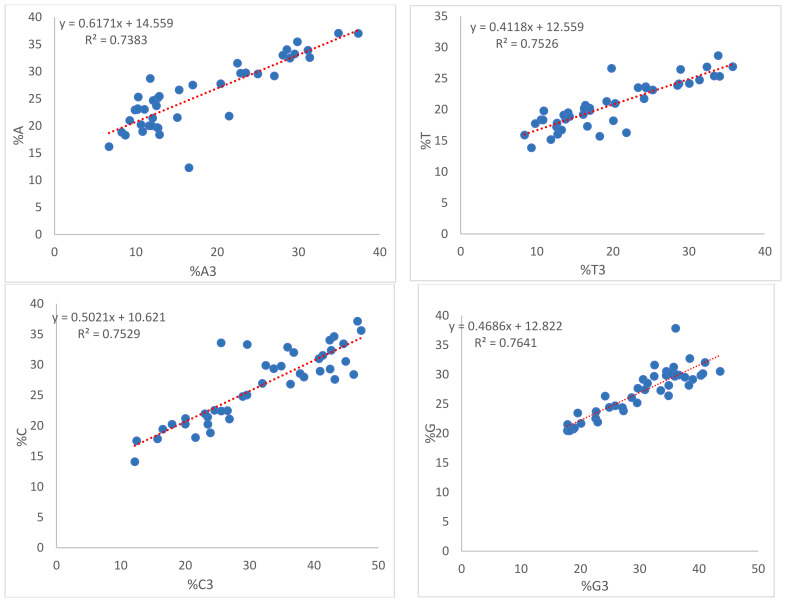
Regression analysis between the overall nucleotide composition and composition at the third position of the codon.

Regression analysis conducted between the overall nucleotide composition and composition at the third codon position (Figure 12) revealed that mutational forces operated on all nucleotides. Nucleotide A was subjected to the greatest mutational forces (61.71%), while T was subjected to the least (41.18%).

### 3.12. Parity Plot Analysis

**Figure 13:**
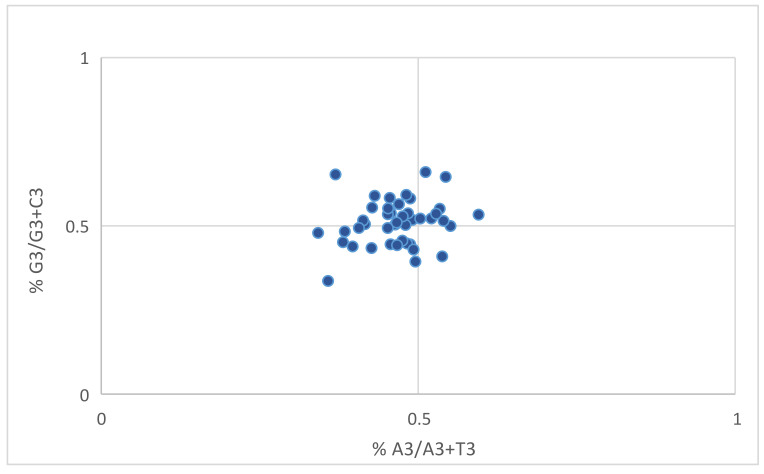
Parity plot analysis depicting preference of T and G over A and C.

Parity plot analysis provides insight into the effects of evolutionary forces on codon usage [31]. Chargaff’s rule dictates that the number of A=T and C=G (Sueoka, 1999) [32]. At the center of the plot, the value of the average position of x = 0.466 ± 0.055 (AT bias) and y = 0.509 ± 0.067 (GC bias) (Figure 13). This result reveals a preference for T and G over A and C.

### 3.13. Relation of Protein Indices with CAI-18

**Figure 14:**
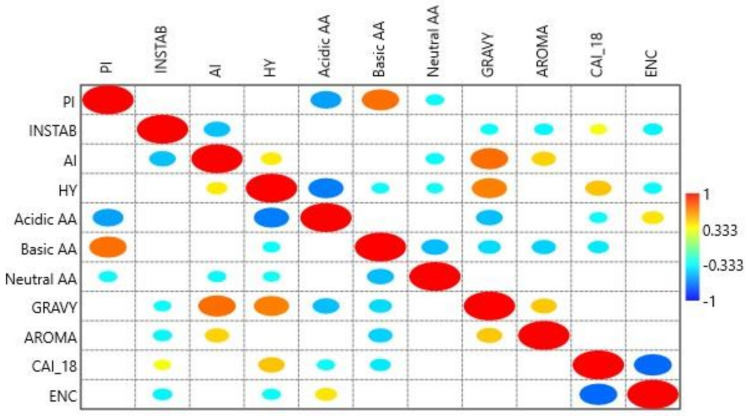
A mirror image plot depicting the correlation between CAI-18 and different protein indices. A bigger-sized eclipse described a higher Pearson’s correlation coefficient (r) value, and vice versa. The red color shows a positive correlation, whilst blue indicates a negative correlation. Empty boxes show an insignificant correlation.

A correlation was determined between CAI-18 and ENc and various protein characteristics including GRAVY, AROMA, PI, AI, HY, INSTAB, and acidic, basic, and neutral AAs (Figure 14). The analysis revealed CAI-18 was positively correlated with the insatiability index (r = 0.309; *p* < 0.05) and hydrophobicity (r = 0.490; *p* < 0.01) but negatively correlated with acidic (r = −0.327; *p* < 0.05) and basic amino acid residues (r = −0.382; *p* < 0.05). ENc was negatively correlated with the insatiability index (r = −0.356; *p* < 0.05) and hydrophobicity (r = −0.334; *p* < 0.05), and positively correlated with acidic AAs (r = −0.403; *p* < 0.01). Protein length did not correlate with any of the protein indices, CUB, or gene expression (data not presented).

## 4. Discussion

Compositional analysis of the selected genes revealed an overall lower occurrence of the T nucleotide. GC3 composition varied the most among all GC positions due to its neutral position on the codon; mutations at this position are silent and do not alter what the codon represents with regard to the amino acid coded. It indicates the action of mutational force in determining the composition of the gene.

In proteins 150 to 3000 AAs in length, the GC3 content was higher than GC12 without exception. Proteins less than 150 AAs and more than 3000 AAs showed higher GC12. The protein length affects several parameters, including folding enthalpy, entropy, and heat capacity [33]. Furthermore, protein stability depends on the chain length and numbers of acidic and basic side chains [34,35]. Hence, to retain optimum stability, the PID gene protein chain length ranged between 150 and 1500 AAs (Figure 3). The abundance of GC3 content is consistent with results obtained by O’Connell et al. (2012), who reported gene expression depended upon the abundance of G and C at the third codon position (GC3) in the genome of *Arabidopsis thaliana* [36]. Also similar to O’Connell et al. (2012), we observed a significantly high positive correlation between the CAI-59 and GC3 contents (*p* < 0.0001). The lack of a correlation between CAI-59 and GC2 and GC12 and a positive correlation with GC1 (*p* < 0.05) cumulatively suggest that at non-neutral positions, the composition plays a minor role in affecting gene expression. The GC components at all positions were inversely correlated with ENc. Hence when the GC3 component increased, CUB also increased [37]. Concerning the CUB and individual codon position, the analysis revealed that CUB was independent of T and G nucleotide compositions at the first codon position (Table 4).

Protein length and CUB have been found to be negatively correlated in *Saccharomyces cerevisiae*, and highly expressed proteins also tend to be of smaller size [38]. A positive correlation was found between CUB and *Drosophila melanogaster*, results opposite to those found for *Saccharomyces cerevisiae*. Energetically costly, longer genes have higher CUB values to maximize translational efficiency. Both positive and negative correlations can be understood based on selection [39]. However, in the present study, protein length did not correlate with any of the protein indices, CUB, or gene expression (data not presented).

The four standard DNA nucleotides can form 16 combinations of dinucleotides. These dinucleotides do not appear in numbers as expected, and few dinucleotides are underrepresented relative to others [40]. Our correlation analysis between the 16 dinucleotide odds ratio and gene expression with CUB revealed that of the two dinucleotides TpA and CpG, which are considered to be under extreme selection pressure, TpA negatively affected protein expression, while CpG did not [26]. It has been shown for the human immunodeficiency virus type 1 *gag* gene that codon optimization resulted in enhancement of CpG rich sequences and had a high level of expression compared to non-optimized sequences [41]. In contrast, CpG dinucleotides are statistically underrepresented in eukaryotes because CpG dinucleotides are often methylated at the fifth position of cytosine and subsequently prone to deamination, resulting in the formation of thymidine out of cytosine [42]. CpG-dense areas are also present in the genome and are mainly confined within or near promoter regions. Furthermore, these CpG-dense regions are protected from CpG methylation through the occupation of the regions by nuclear factors [43]. Bauer et al. (2010) revealed the influence of intragenic CpG content on gene expression by comparing the expression of a GFP reporter in a codon-optimized CpG-depleted version with its CpG-rich equivalent [44]. The GFP variant lacking CpG led to reduced GFP reporter expression irrespective of the cell type and promoter used. In the present study, the CpG dinucleotide had no impact on PID gene expression but negatively affected CUB.

Dinucleotide TpA negatively affects gene expression in PID genes, and possibly due to being a part of stop codons TAA, TAG, and TGA, leads to the premature termination of the growing protein chain. ENc was positively correlated with TpA dinucleotides, showing that with an increase in TpA content, CUB decreased [17].

In PID genes, codons ending in CG were preferred over codons ending in AT (Figure 5). This pattern was not followed by *CASP, FAS, ATM, BLM, NBN, KRAS, PIK3R1, NRAS, MALT1, PTPRC, IL7R, SBDS, and PMS2* genes, in which codons ending in AT were preferred over GC-ending codons, observed in the richness of A and T nucleotides in the overall composition of these genes. Our results indicate the influence of composition on codon usage preference. Furthermore, as we found no effect of the CpG dinucleotide on protein expression (Figure 4), it is obvious to expect no effect of CpG-containing codons on gene expression. This assumption was partially correct as five codons—CGA, CGT, CCG, TCG, and GCG—did not correlate with either gene expression or CUB, while three codons—CGG, ACG, and CGC—positively correlated with gene expression and negatively correlated with CUB. Moreover, CUB did not correlate with %T1 and %C1 but correlated with nucleotide compositions at other codon positions to varying degrees (*p* < 0.5 to *p* < 0.0001) (Table 4). All of these assertions indicate that not only the compositional properties but also other factors influence gene expression and CUB.

Codon CTG was overrepresented with a very high RSCU value in most genes and negatively correlated with the AGA codon (Figure 6a). The CTG codon was overexpressed (overrepresentation, RSCU > 1.6) in 80.95% of genes, underexpressed (underrepresentation, RSCU = 0) in 2.38% of genes, and randomly used (RSCU >0.6 and <1.6) in 16.66% of genes. The CTG codon has been found overexpressed in obesity, housekeeping [45], and central nervous system genes [46]. In Y-linked genes, the RSCU values indicated overrepresentation of both AGA and CTG codons [47]. In PID genes, all three AGA and CTG codon representation scenarios were observed. In a few PID genes, the AGA codon was highly underrepresented while CTG was overrepresented (17 genes) [Figure 6a], while in a few genes, the reverse occurred (one gene with underrepresentation of CTG and overrepresentation of AGA). Some genes showed overrepresentation of both AGA and CTG (Figure 6b). Runs of AGA codons have been linked to translational regulation by tRNA methyltransferase [48]. Hence, the number of AGA codons utilized appears tightly linked with transitional regulation and selection pressure.

ENc-GC3 curve analysis (Figure 10) demonstrated that selectional pressure was strongly involved with mutational forces. A significant positive correlation was observed between overall nucleotide composition and nucleotide composition at the third position of the codon in homologous and heterologous nucleotides. Furthermore, a negative correlation observed between overall nucleotide composition and nucleotide composition at the third codon position in heterologous nucleotides indicates the role of mutational forces on CUB [49]. Overall, selectional forces contributed 72.82% of the variation, while mutational forces contributed 31.6% of the variation (Figure 11). Mutational forces at the individual nucleotide level were observed the most in nucleotide A (61.71%), while nucleotide T experienced the least (41.18%) (Figure 12).

When selection and mutational forces are equal, on a parity plot, all the points will be positioned in the center where A=T and C=G. The parity plot can distinguish between AT and GC bias [50]. All the nucleotides were not used equally in the PID genes, and T and G were preferred over A and C.

Various protein characteristics were evaluated for their correlation with gene expression and CUB. The instability index refers to the protein stability under both in vivo and in vitro conditions. Proteins with instability indices >40 are considered unstable, while those <40 are stable [51]. Instability index values ranged from 27.89 (FAS) to 70.11 (WAS). The instability index was positively correlated with CAI-18 and negatively correlated with ENc, indicating that gene expression and CUB increase with an increasing instability index.

Hydrophobic interactions are the forces that keep the protein structure together. Molecules with similar hydropathy have an affinity for each other and are compatible and interact [52]. Hydrophobic bonds in a protein contribute to the formation of the protein’s native structure and nucleation site to initiate protein folding [53]. Hydrophobicity positively correlated with CAI-18 and negatively correlated with CUB, indicating that hydrophobicity promotes both protein expression and CUB.

Protein expression and CUB significantly correlated with the number of acidic amino acid residues present in the protein. A positive association with protein expression was found for basic AAs with no impact on CUB, cumulatively signifying their role in contributing to selection pressure.

## 5. Conclusions

The immune system is a set of highly specialized cells, tissues, and organs that together provide an organism’s immunity. A faulty immune system with dysregulation in the humoral, cellular, or both arms of the immune system might result in developmental disorders, autoimmunity, inflammatory disorders, recurrent infections, and increased cancer incidence. Initial evidence of the linkage between cancer and PIDs was revealed in 1958, and the genetic basis of PIDs and cancer is based mainly on monogenic disorders. To date, more than 350 genes have been identified as contributing to PIDs. The close association of PIDs and cancer prompted researchers to investigate their common features at the genetic level. In the present study, a panel of 42 genes shared in both PIDs and cancer were investigated for codon usage bias, nucleotide composition, protein properties, and expression profiling to gain in-depth knowledge of these genes.

The T nucleotide was generally present at a low level in these genes, and %GC3 was the most variable among all %GC positions. The %GC3 was high for all the genes except those smaller than 150 amino acids and larger than 3000 amino acids, without exception. In the present study, no correlation between gene length and gene expression was observed. The dinucleotides TpA and CpG experienced the highest selectional force. TpA negatively correlated with gene expression, while CpG had no impact on gene expression. Regarding CUB, increased CpG resulted in an increase in bias, and the opposite was true for TpA. Codon CTG was the most overexpressed in most genes (80.95%), with random usage in few genes (16.66%).

ENc-GC3 curve analysis, parity analysis, and neutrality analysis revealed that selection pressure, mutation pressure, and compositional constraints are all responsible for shaping codon bias, and the role of selection force is dominant. Among all nucleotides, T was subjected to the least mutational force. Analysis of various protein properties influencing codon usage, including GRAVY, AROMA, isoelectric point, aliphatic index, hydrophobicity index, instability index, numbers of acidic amino acid residues, basic amino acid residues, and neutral amino acid residues revealed that hydrophobicity index, instability index, and numbers of acidic and basic amino acid residues were the factors affecting gene expression. Hydrophobicity index, instability index, and numbers of acidic amino acids were the factors affecting CUB.

To summarize, selection pressure was the primary force. Selection favored few protein characteristics such as hydrophobicity index, instability index, and numbers of acidic and basic amino acid residues and codon CTG in order to facilitate the protein expression in genes common to PIDs and cancer.

Overall, in present analysis, we tried to investigate various compositional parameters, CUB, effects of various parameters on CUB including length, physical properties of proteins, mutational, selectional, and other forces, and upon analysis, it was determined that selection pressure, mutation pressure, and compositional constraints all participated in shaping codon usage.

## Figures and Tables

**Table 1:** Panel of 42 of genes common to both PIDs and cancer.

	Gene Name										
1	*NBN*	8	*PRF1*	15	*JAK3*	22	*STAT5B*	29	*CD79A*	36	*ATM*
2	*CARD11*	9	*STAT3*	16	*LCK*	23	*CDKN2A*	30	*CD79B*	37	*BLM*
3	*CASP8*	10	*PIK3R1*	17	*MALT1*	24	*CSF3R*	31	*NFKB2*	38	*FCGR2B*
4	*FAS*	11	*CIITA*	18	*MSN*	25	*WAS*	32	*PMS2*	39	*IKZF1*
5	*ITK*	12	*IKBKB*	19	*PTPRC*	26	*GATA2*	33	*TCF3*	40	*POLE*
6	*KRAS*	13	*IL21R*	20	*RECQL4*	27	*SBDS*	34	*CXCR4*	41	*TERT*
7	*NRAS*	14	*IL7R*	21	*RHOH*	28	*BTK*	35	*MYD88*	42	*TNFRSF1A*

**Table 2:** Correlation analysis between overall GC content and GC components at different positions along with CAI-59 and ENc.

	%GC	%GC1	%GC2	%GC12	%GC3	CAI_59	ENc
%GC		<0.0001	<0.0001	<0.0001	<0.0001	<0.0001	<0.0001
%GC1	0.8757		<0.0001	<0.0001	<0.0001	<0.05	<0.001
%GC2	0.7853	0.7813		<0.0001	<0.01	NS	<0.001
%GC12	0.8796	0.9428	0.9447		<0.0001	NS	<0.001
%GC3	0.8880	0.6109	0.4519	0.5624		<0.0001	0.0001
CAI_59	0.6586	0.3343	0.1662	0.2644	0.8893		<0.0001
ENc	−0.7394	−0.5100	−0.5382	−0.5554	−0.7484	−0.6530	

The red color font shows the negative correlation and black colored font shows positive correlation.

**Table 3:** Effect of nucleotides in agreement to their position in codon on CUB.

ENc	%A	%C	%T	%G	%A1	%C1
Pearson’s r value	0.70745	−0.72116	0.6693	−0.65231	0.55162	−0.52466
*p* value	<0.0001	<0.0001	<0.0001	<0.0001	<0.001	<0.001
ENc	%T1	%G1	%A2	%C2	%T2	%G2
Pearson’s r value	0.25318	−0.22447	0.51862	−0.46184	0.30778	−0.49723
*p* value	NS	NS	<0.001	<0.01	<0.05	<0.001
ENc	%A3	%C3	%T3	%G3	%GC	%GC1
Pearson’s r value	0.74303	−0.70233	0.7093	−0.64765	−0.73937	−0.50996
*p* value	<0.0001	<0.0001	<0.0001	<0.0001	<0.0001	<0.001
ENc	%GC2	%GC12	%GC3	%GC3	--	--
Pearson’s r value	−0.53816	−0.55542	−0.74837	−0.74837		
*p* value	<0.001	<0.001	<0.001	<0.0001		

NS, non significant.

**Table 4:** Correlation analysis for overall nucleotide composition with composition at third codon position.

	%A	%C	%T	%G	%G+C
%A3	0.859 ***	−0.852 ***	0.725 ***	−0.745 ***	−0.862 ***
%C3	−0.796 ***	0.868 ***	−0.702 ***	0.601 ***	0.811 ***
%T3	0.772 ***	−0.823 ***	0.868 ***	−0.78 ***	−0.862 ***
%G3	−0.719 ***	0.667 ***	−0.799 ***	0.874 ***	0.799 ***
%G3+C3	−0.841 ***	0.863 ***	−0.818 ***	0.788 ***	0.888 ***

Negative correlations are depicted with red colored font while positive correlations have been depicted with black font. *** *p* < 0.0001.

## Data Availability

Available upon request.

## Supplementary Materials

The following are available online at https://www.mdpi.com/article/10.3390/biomedicines9081001/s1, Table S1: The RSCU values of genes involved in both PIDs and cancer. The codon having highest RSCU value for a given amino acid is highlighted.
